# Low-Dose Dexmedetomidine Accelerates Gastrointestinal Function Recovery in Patients Undergoing Lumbar Spinal Fusion

**DOI:** 10.3389/fphar.2019.01509

**Published:** 2019-12-19

**Authors:** Meng Li, Tianlong Wang, Wei Xiao, Lei Zhao, Dongxu Yao

**Affiliations:** ^1^ Department of Anesthesiology, Beijing Xuanwu Hospital, Capital Medical University, Beijing, China; ^2^ National Clinical Research Center for Geriatric Diseases, Beijing, China

**Keywords:** dexmedetomidine, flatulence, inflammation mediators, spinal fusion, opioids

## Abstract

**Background:** Dexmedetomidine possesses sedative, sympatholytic, and opioid-sparing properties, but its impact on postoperative gastrointestinal function is controversial.

**Methods:** This single-center, prospective, randomized study compared low-dose dexmedetomidine and placebo on gastrointestinal function recovery and inflammation after posterior lumbar spinal fusion. Sixty-six patients were randomized into two groups and received normal saline (control group) or dexmedetomidine (DEX group) during posterior lumbar fusion. Blood was taken at five timepoints to measure lipopolysaccharides, tumor necrosis factor-α, and C-reactive protein. The primary outcome was duration to first flatus. The secondary outcomes were inflammatory mediators and determination of correlations between perioperative factors and duration to first flatus.

**Results:** Patients in DEX group showed significantly lower duration to first flatus (15.37 [13.35–17.38] vs 19.58 [17.31–21.86] h; *p* = 0.006) and overall sufentanil consumption (67.19 [63.78–70.62] vs 74.67 [69.96–79.30] μg; *p* = 0.011) than controls. Lipopolysaccharides, tumor necrosis factor-α, and C-reactive protein did not differ between the groups at any timepoint (all *p >* 0.05). Multiple linear regression modeling assessed the ability of independent variables to predict variance in duration to first flatus (adjusted *R^2^* = 0.379, *p* = 0.000). In the model, age (*β* = 0.243, *p* = 0.003), gender (*β* = −3.718, *p* = 0.011), BMI (*β* = −0.913, *p* = 0.001), operative segments (*β* = −4.079, *p* = 0.028), and overall sufentanil consumption (*β* = 0.426, *p* = 0.000) contributed significantly.

**Conclusions:** Thus, low-dose dexmedetomidine accelerates gastrointestinal function recovery after lumbar spinal fusion. The effect may be partially produced by opioid-sparing effects rather than inhibition of inflammation.

**Clinical Trial Registration:** www.chictr.org.cn, identifier ChiCTR1800018127.

## Introduction

Patients undergoing lumber spinal surgery usually encounter postoperative transient gastrointestinal (GI) dysfunction due to prolonged bed rest. The incidence of postoperative ileus was 26.0 per 1,000 (Fineberg et al., 2014) in posterior lumbar fusions and postoperative ileus was associated with increased length of hospital and costs. Potential mechanisms/factors leading to GI dysfunction may include activation of inhibitory sympathetic reflexes, systematic use of opioids, surgical trauma-induced inflammatory responses, and prolonged bed rest (Bauer and Boeckxstaens, 2004). Accelerating recovery of GI function is important to enhancing overall recovery after surgery as it could reduce the rate of postoperative infection and shorten hospital stays (Yang et al., 2016).

Dexmedetomidine (DEX) is a potent and highly selective α_2_-adrenoreceptor agonist widely used as an anesthetic adjuvant during surgery (Tanskanen et al., 2006). DEX also offers intraoperative anesthetic-sparing, analgesic-sparing, and anti-inflammatory effects (Arcangeli et al., 2009; Dong et al., 2017) and is safe for use with elderly patients. However, the impact of DEX on postoperative GI function is controversial. DEX was revealed to protect intestine from injury caused by intestinal I/R and endoxemia (Kilic et al., 2012; Zhang et al., 2012). Chen et al. (2016) also demonstrated that DEX can benefit recovery of GI motility function after laparoscopic resection of colorectal cancer. Whereas the antiperistaltic effects of DEX were seen *in vitro* (Herbert et al., 2002), animal experiments (Asai et al., 1997) and healthy volunteers (Iirola et al., 2011). The mechanisms underlying DEX’s effect on GI function are also complex. DEX may reduce sympathetic tone and thus promote peristalsis by acting on central α_2_-adrenoceptors (Cho et al., 2015); on the other hand, DEX may activate inhibitory α_1_-adrenoceptors located postsynaptically on the smooth muscle or activate inhibitory α_2_-adrenoceptors on excitatory cholinergic pathways to inhibit peristalsis (De Ponti et al., 1996). The inhibitory effects of DEX on GI motility were likely to be dose-dependent (Iirola et al., 2011). This study thus employed a least recommended clinical dose of DEX to reduce the side effects and ensure safety in elderly patients. Based on the characteristics of DEX and the possible mechanisms underlying postoperative GI dysfunction, the present study assumed that administration of this low-dose DEX during lumbar spinal fusion surgery may accelerate postoperative GI function recovery and/or attenuate inflammation. This study compared low-dose dexmedetomidine and placebo on gastrointestinal function recovery and inflammatory mediators after posterior lumbar spinal fusion. The study also analyzed correlations between perioperative factors and duration to first flatus.

## Methods

### Study Design

This single center trial has a prospective, randomized parallel-group design. The present study was conducted at Xuan Wu Hospital, Capital Medical University (Beijing, China) from September 1, 2018, to March 1, 2019. The hospital’s Ethics Committee approved the study protocol (2017–076), which was performed in accordance with the Declaration of Helsinki. After approval, this study was registered in the Chinese Clinical Trial Registry (website: www.chictr.org.cn, ChiCTR1800018127) and adhered to CONSORT guidelines. Verbal and written informed consents were obtained from all the included patients.

### Patients

Patients with lumbar disk herniation or lumbar spondylolisthesis undergoing elective lumber spinal surgery were enrolled into this study. The type of surgery was posterior lumbar discectomy + pedicle screw fixation +intertransverse fusion. The inclusion criteria were an American Society of Anesthesiologists physical status below 4 and patients aged 18 years or older. The exclusion criteria included patient refusal to participate at any time, preoperative use of opioid, bradycardia (heart rate < 50 bpm), heart block greater than the first degree, preoperative use of antihypertensive drugs containing clonidine or an α_2_-adrenergic receptor agonist, difficulty in communication, abnormal liver or renal function, and bowel disease (e.g., ulcerative colitis, Crohn’s disease, and irritable bowel syndrome).

### Randomization and Blinding

The included patients were randomized into two groups with an allocation ratio of 1:1 and either received normal saline (control group) or low-dose DEX (DEX group) during surgery. Randomization was based on online randomization software (https://tools.medsci.cn/rand)—generated codes stored in sequentially numbered and sealed envelopes. The study agents (normal saline and DEX) were prepared in identical 50-ml syringes by a research nurse who was blinded to group assignments. The randomization code was broken only after patient enrollment and follow-up had ended. All surgeons, patients, attending anesthesiologists, nurses, and follow-up anesthesiologists were blinded to group assignments.

### Interventions

The anesthesia management protocol was standardized among the groups. All patients fasted for 12 h and were given soapsuds enemas the night before surgery. Heart rate, invasive blood pressure, pulse oxygen saturation, nasopharyngeal temperature, end-tidal carbon dioxide level, and bispectral index (BIS, Aspect A-2000; Aspect Medical Systems Inc., Newton, MA, USA) were routinely monitored. Goal-directed fluid therapy was used intraoperatively to maintain pulse pressure variation below 13%. The maintenance fluid infusion rate was set at 1–2 ml/kg/h. Anesthesia was induced with etomidate (0.2–0.3 mg/kg), sufentanil (0.2–0.3 µg/kg), and rocuronium (0.6–0.8 mg/kg). Volume-controlled ventilation was performed with an oxygen-air mixture (fraction of inspired O_2_, 0.5) to maintain an end-tidal CO_2_ between 30 and 35 mm Hg. Total intravenous anesthesia was maintained with continuous infusion of remifentanil (0.3–0.4 µg/kg/min), propofol (3–6 mg/kg/h), and cisatracurium (1–2 µg/kg/min). The propofol infusion rate was hand-titrated to maintain a bispectral index value between 40 and 60. Additional sufentanil was added in 10-µg increments at the skin incision as needed as well as 30 min before surgical suture.

DEX group patients were given dexmedetomidine (4 μg/ml) continuously after turning to the prone position; the loading dose was 0.5 μg/kg for 15 min and was then maintained at a rate of 0.1 μg/kg/h until 30 min before skin suture. The control group underwent the same procedure but received saline instead.

The day before surgery, all patients were instructed on the use of a 10-point numeric rating scale to assess their pain intensity (0 = no pain, 10 = worst possible pain). Postoperative patient-controlled intravenous analgesia was performed with sufentanil (1.5 μg/kg). All patients received a basal dose of (0.015 μg/kg/h) and PCA (0.03 μg/kg). The interval time was set at 10 min, and patient-controlled intravenous analgesia was maintained up to 72 h following surgery to make sure all patient numeric rating scale scores were below 3.

### Blood Samples

Blood samples (3 ml each) were taken from a peripheral vein at the following timepoints: before induction (baseline; T_0_), at the end of surgery (T_1_), and 24 h (T_2_), 48 h (T_3_), and 72 h (T_4_) after surgery. All blood samples without anticoagulation were centrifuged at 3000 rpm for 15 min to collect serum, which was frozen at −80°C. Enzyme-linked immunosorbent assay kits were used to measure serum concentrations of the inflammatory mediators C-reactive protein (CRP; Biokits Tech Inc., Beijing, China), tumor necrosis factor-α (TNF-α; Biokits Tech Inc.), and lipopolysaccharides (LPS; Nanjing Jiancheng Bioengineering Institute Inc., Nanjing, China) according to the manufacturers’ instructions. All samples were analyzed at a dilution resulting in concentrations within the range of the standard curve.

### Data Collection

Demographic data, including age, gender, body mass index, diabetes mellitus, constipation, and American Society of Anesthesiologists level, were recorded. The mean blood pressure and heart rate were monitored and recorded at baseline (before induction), before intervention, immediately after loading dose, and at the end of surgery. The overall consumption of DEX and narcotics, surgical indices, including operative segments, anesthesia duration, surgical duration, blood loss, urine output, and fluid infusion amount, were recorded. Duration to first flatus and needs for blind enema were recorded during follow-up.

The primary outcome of the present study was duration to first flatus, which was self-reported by patients and recorded by a follow-up anesthesiologist who was blinded to group assignments during follow-up. The secondary outcomes were inflammatory mediators and determination of any correlations between perioperative factors and duration to first flatus. Inflammatory mediators were assessed at the following timepoints: before induction (baseline; T_0_), at the end of surgery (T_1_), and 24 h (T_2_), 48 h (T_3_), and 72 h (T_4_) after surgery.

### Statistical Analysis

According to the results of a preliminary study (*n* = 20), the duration to first flatus was 21.1 h [5.8 h] in the DEX group and 25.6 h [8.3 h] in the control group. This meant that a sample size of at least 33 was needed in each group to have a power of 95% to detect differences of 10% or more between the groups. Therefore, a total of 90 patients were initially recruited in the present study to compensate for any exclusion and ensure a minimum sample size of at least 33 patients per group.

Statistical analysis was done with SPSS software version 17.0 (SPSS Inc., Chicago, IL, USA). All quantitative data are presented as means ± standard deviations, with intergroup comparisons made by unpaired Student’s *t*-test and intragroup comparisons done by repeated measures one-way analysis of variance. The median (interquartile range [IQR]) of rank data and non-normally distributed quantitative variables are also presented, with intergroup comparisons made with the nonparametric Mann–Whitney test. Qualitative variables are expressed as percentages and were compared *via* Chi-squared test. Multiple linear regression was performed to assess the ability of perioperative factors to predict variances in duration to first flatus. A *p* < 0.05 was considered statistically significant.

## Results

**Figure 1:**
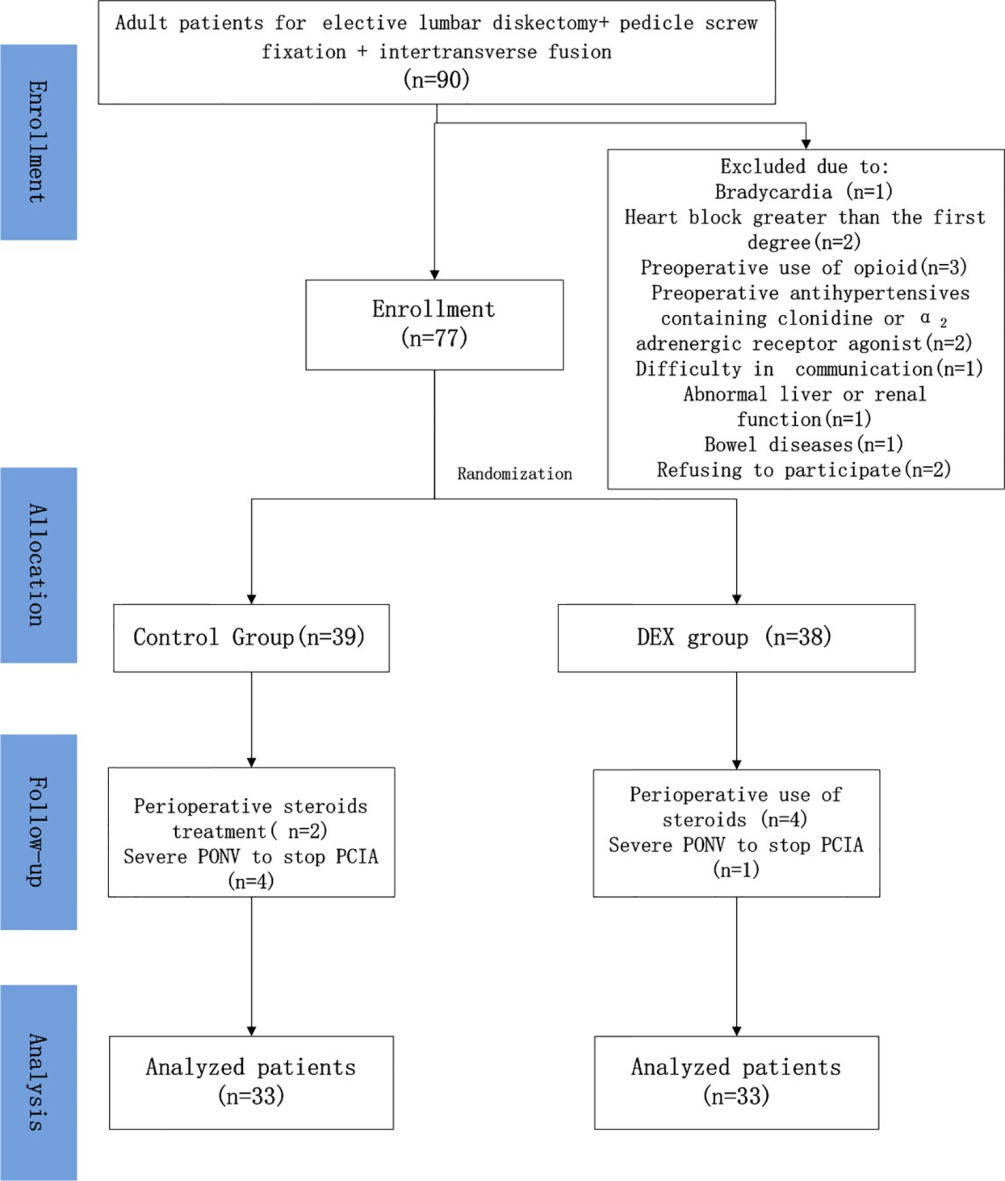
Patient recruitment flowchart. Patients were excluded from the study due to bradycardia (*n* = 1), heart block greater than the first degree (*n* = 2), preoperative use of opioids (*n* = 3), preoperative use of antihypertensives containing clonidine or an α_2_-adrenergic receptor agonist (*n* = 2), difficulty in communication (*n* = 1), abnormal liver or renal function (*n* = 1), bowel disease (*n* = 1), and refusal to participate (*n* = 2). Six patients in the control group and five in the dexmedetomidine (DEX) group were excluded due to perioperative use of steroids or severe postoperative nausea and vomiting that required stopping patient-controlled intravenous analgesia (PCIA). ASA, American Society of Anesthesiology.

Of the 90 patients initially assessed for eligibility, 13 met the exclusion criteria. The remaining 77 patients were randomized into the DEX (*n* = 38) and control (*n* = 39) treatment groups. After exclusion of an additional 11 patients, data from a total of 66 patients (*n* = 33 per group) were included in the analysis (**Figure 1**).

Patient age, gender, body mass index, diabetes mellitus, constipation, American Society of Anesthesiologists level, as well as pre- and postoperative white blood cell counts were similar between the treatment groups (**Table 1**). There were no significant differences in operative time, operative segments, anesthesia duration, surgical duration, blood loss, fluid infusion amount, urine output, overall remifentanil consumption or needs for blind enema (**Table 1**). The incidence of intraoperative bradycardia in the DEX group was significantly lower than in the control (12.12% versus 36.36%, *p* = 0.021; **Table 1**). The overall sufentanil consumption in the DEX group was significantly less than in the control group [67.19 (63.78–70.62) vs 74.67 (69.96–79.30) μg; *p* = 0.011; **Table 1**].

**Table 1:** Patients characteristics.

Characteristics	Control Group (n = 33)	Dex Group (n = 33)	*p value*
Age, years	60.91 (8.75)	59.27 (9.25)	0.463
Gender, female/n	60.61	57.58	0.802
BMI, kg/m^2^	26.42 (3.65)	25.45 (3.28)	0.263
ASA level	2( 2–2)	2( 2–2)	0.286
Diabetes mellitus, %	21.21	15.15	0.750
Constipation, %	0	3.03	1.000
Preoperative WBC counts, 10^9^/L	6.20 (1.36)	6.31 (1.37)	0.741
Postoperative WBC counts, 10^9^/L	9.17 (2.80)	9.14 (2.64)	0.973
Operative segments	2 (1–2)	2 (1–2)	0.939
Ratio of single operative segment, %	45.45	48.48	1.000
Anesthesia duration, min	269.24 (59.94)	249.36 (51.33)	0.153
Surgical duration, min	203.12 (47.72)	190.21 (50.61)	0.290
Blood loss, ml	300 (150–400)	240 (150–475)	0.684
Fluid infusion amount, ml	1441.52 (348.21)	1403.64 (336.09)	0.654
Urine output, ml	824.24 (425.76)	915.15 (394.59)	0.372
HR < 50, beat/min	36.36	12.12	0.021
Overall consumption of sufentanil, μg	74.67 (13.19)	67.19 (9.64)	0.011
Overall consumption of remifentanil, mg	5.1 (1.5)	4.7 (1.6)	0.244
Overall consumption of dexmedetomidine, μg	0	51.84 (12.10)	<0.0001
Needs for blind enema, %	12.12	3.03	0.355

Data were expressed as mean (SD), median (interquartile range), or n (%).

Dex, dexmedetomidine; BMI, body mass index; ASA, American Society of Anesthesiologist; WBC, White blood cell.

The duration to first flatus was significantly shorter in the DEX group than in the control group [15.37 (13.35–17.38) vs 19.58 (17.31–21.86) h; *p* = 0.006].

Baseline (T_0_) serum LPS was obviously higher than the normal limit (<54.2 EU/L) in both treatment groups. While the T_1_–T_4_ LPS levels in the DEX group were all significantly lower than that at T_0_ (all *p* < 0.05), the control group levels did not begin to significantly decrease until T_2_ (**Table 2**). Compared with T_0_ levels, serum TNF-α significantly increased at T_2_–T_4_ in both treatment groups (*p <* 0.05; **Table 2**). Serum CRP levels at T_2_–T_4_ were significantly higher than those at T_0_ in both treatment groups (*p <* 0.05; **Table 2**). However, there were no apparent differences in inflammatory mediators between the treatment groups at any timepoints (**Table 2**).

**Table 2:** Perioperative inflammatory mediators.

Inflammatory mediators	Timepoints	Control Group (n = 33)	Dex Group (n = 33)	*p* value
LPS, EU/ml	T_0_	135.1 (79.6)	125.5 (85.2)	0.638
	T_1_	120.8 (77.4)	94.8 (58.6)*	0.366
	T_2_	103.5 (61.1)*	90.5 (55.6)*	0.509
	T_3_	100.1 (61.4)*	80.5 (39.8)*	0.133
	T_4_	99.2 (53.8)*	75.3 (41.9)*	0.076
TNF-α, pg/ml	T_0_	15.13 (10.56)	16.37 (12.71)	0.944
	T_1_	15.18 (10.14)	16.35 (12.52)	0.928
	T_2_	62.68 (39.56)*	59.55 (30.33)*	0.969
	T_3_	74.89 (39.80)*	72.81 (40.53)*	0.663
	T_4_	64.71 (37.50)*	61.26 (37.22)*	0.847
CRP, ng/ml	T_0_	454.2 (344.3)	465.8 (407.0)	0.901
	T_1_	386.9 (308.1)	403.7 (359.4)	0.686
	T_2_	13702 (2090)*	12972 (2030)*	0.155
	T_3_	14298 (2099)*	13543 (2334)*	0.162
	T_4_	13706 (3111)*	12576 (3263)*	0.079

Data were expressed as mean (SD). *compared with T_0_, P < 0.05; P in the table indicated the significant differences between two groups.

Dex, dexmedetomidine; LPS, lipopolysaccharides; TNF-α, tumor necrosis factor-α; CRP, C-reactive protein; T_0,_ baseline before induction; T_1,_ at the end of surgery; T_2,_ 24 _h_ after surgery; T_3,_ 48 _h_ after surgery; T_4,_ 72 h after surgery.

Based on clinical considerations, the multiple linear regression model established to assess the ability of selected independent variables (age, gender, BMI, overall sufentanil consumption, surgical duration, and operative segments) to predict variance in duration to first flatus was statistically significant (adjusted *R^2^* = 0.379, *p* = 0.000). Gender (female, 1; male, 2) and operative segments (single segment, 1; more than one segment, 2) were analyzed as categorical variables in the multiple linear regression analysis. In the model, age (*β* = 0.243, *p* = 0.003), gender (*β* = −3.718, *p* = 0.011), BMI (*β* = −0.913, *p* = 0.001), operative segments (*β* = −4.079, *p* = 0.028), and overall sufentanil consumption (*β* = 0.426, *p* = 0.000) contributed significantly (**Table 3**).

**Table 3:** Correlation coefficient and standard error of selected independent variables and duration to first flatus.

Screened Variables	Correlation coefficients (β)	Standard error	*p* value
Age	0.243	0.078	0.003
Gender	−3.718	1.411	0.011
BMI	−0.913	0.258	0.001
Surgical duration	−0.017	0.018	0.339
Operative segments	−4.079	1.815	0.028
Overall consumption of sufentanil	0.426	0.075	0.000

BMI, body mass index.

In general, the mean blood pressure and heart rate were similar between the two groups (**Table 4**). However, the mean blood pressure at the end of surgery in the DEX group trended to be lower than in the control group, though not significantly (*p* = 0.064; **Table 4**).

**Table 4:** Intraoperative hemodynamic and respiratory parameters.

		Control Group (n = 33)	Dex Group (n = 33)	*p value*
MAP, mmHg	T_1_	100.55 (10.45)	100.48 (9.32)	0.980
	T_2_	91.67 (10.02)	93.03 (8.26)	0.548
	T_3_	92.15 (9.85)	96.30 (11.44)	0.119
	T_4_	91.54 (8.80)	87.55 (8.46)	0.064
HR, beat/min	T_1_	73.48 (10.90)	75.82 (9.26)	0.352
	T_2_	61.82 (10.51)	65.36 (6.74)	0.108
	T_3_	57.27 (8.65)	59.72 (6.72)	0.203
	T_4_	65.21 (11.89)	64.85 (6.40)	0.878

Data were expressed as mean (SD).

Dex, dexmedetomidine; MAP, mean arterial pressure; HR, heart rate; T_1_, baseline (before induction); *T_2_,* before intervention; T_3,_ immediately after loading dose; T_4_, at the end of surgery.

## Discussion

Although lumbar spinal fusion surgery does not directly involve the GI tract, postoperative transient GI dysfunction, including delayed defecation, abdominal distention, even ileus can occurs. Various mechanisms/factors have been proposed to explain this dysfunction (Holte and Kehlet, 2000), including surgical trauma, activation of inhibitory sympathetic reflexes, induction of local and systemic inflammatory mediators, systematic opioid use and prolonged bed rest. To improve the quality of postoperative recovery and shorten hospital stays, many efforts are made to facilitate the early return to normal GI function.

DEX is a highly selective α_2_-adrenoceptor agonist with hypnotic, sedative, sympatholytic, and opioid-sparing properties that does not cause respiratory depression (Weerink et al., 2017). However, the impact of DEX on postoperative GI function is controversial. Memis et al. (2006) reported that administration of DEX to critically ill patients at 0.2 μg/kg/h for 5 h had no impact on gastric emptying, while Cho et al. (Cho et al., 2015) indicated that infusion of DEX at 0.4 μg/kg/h after a loading dose of 0.5 μg/kg for 10 min can shorten the duration to first flatus in patients undergoing laparoscopic gastrectomy. On the other hand, Iirola et al. (2011) showed that DEX infused at 0.7 μg/kg/h after a loading dose of 1 μg/kg for 20 min can inhibit gastric emptying in healthy volunteers. Hence, first explanation of the contradictory results is that the inhibitory effects of DEX on GI motility are likely dose-dependent. Herbert et al. (2002) revealed that dexmedetomidine concentration-dependently inhibited peristalsis of the guinea pig ileum *in vitro*, and the inhibition is caused by interaction with α_2_-adrenoceptors. While *in vivo*, sympathetic hyperactivity is also considered as an important factor in the development of postoperative GI atonia (Cho et al., 2015). Low-dose DEX may thus promote peristalsis by acting on central α_2_-adrenoceptors to reduce sympathetic tone (Cho et al., 2015). High-dose DEX may inhibit peristalsis by activating inhibitory α_1_-adrenoceptors located postsynaptically on the smooth muscle or by activating inhibitory α_2_-adrenoceptors on excitatory cholinergic pathways in the enteric nervous system, such as opioid, purinergic, and nitrergic neurons (De Ponti et al., 1996). Compared to Cho et al. (2015), the present study employed an even lower dose of DEX to reduce the side effects and ensure safety in elderly patients, and still achieved promotion of GI recovery.

DEX can produce antinociception *via* activation of central α_2_-adrenoceptors (Yeh et al., 2012), and probably attenuated the effects of surgical stress and pain in surgeries. DEX can also reduce sympathetic nervous activity and result in vasodilation of small vessels (Talke et al., 1997; Bekker et al., 2008). Thus, second explanation of the contradictory results is that DEX may affect GI function differently according to varied status. On physiological status, DEX might inhibit the motility of GI by an effect on enteric neurons (Herbert et al., 2002; Iirola et al., 2011). Whereas on pathological conditions, under which intestinal muscular hypotension can disturb intestinal motility (Overhaus et al., 2004), DEX may improve the GI function by its global hemodynamic stability effect, which can prevent the violent alteration of GI microcirculation, attenuate intestinal I/R injury, and improve stress response (Kilic et al., 2012; Zhang et al., 2012).

Opioids are considered as modulators of transmission in the central and peripheral nervous systems, leading to inhibition of gastric emptying and nonpropulsive smooth muscle contraction with an increase in intraluminal pressure throughout the GI tract (Holte and Kehlet, 2000). This effect is predominantly mediated by mu-opioid receptor agonists. The analgesic and opioid-sparing effects of DEX are thought to be mediated by α_2_-adrenoreceptor binding in the central nervous system and spinal cord α_2_-adrenoreceptors (Weerink et al., 2017). The current results demonstrated the postoperative opioid-sparing effects of DEX, and the overall consumption of sufentanil was significantly positively correlated with duration to first flatus. This suggests that opioid-sparing effects of DEX may partially reduce opioid-induced inhibition of GI motility and shorten the duration to first flatus.

LPS is a product of gut microbiota, and about 100 trillion gut bacteria contribute to an enteric reservoir greater than 1 g of LPS (Pastori et al., 2017). In some clinical settings, such as intestinal ischemia, surgical stress or intestinal mucosa injury, LPS may enter the systemic circulation as a consequence of increased gut permeability. In the present study, a high level of baseline LPS may have been due to soapsuds enema-induced intestinal injury. Bacterial LPS has long been considered to be a main stimulant triggering TNF-α production (Zelova and Hosek, 2013). TNF-α is an important cytokine that can timely, sensitively, and predictably reflect the state of immune function and inflammation after surgical trauma. TNF-α regulates the release of cytokines and CRP through nuclear factor-κB activation. CRP is used to monitor the postoperative course in surgical trauma (Haraguchi et al., 2017). Its serum concentration is very low in healthy subjects but increases rapidly in cases of inflammation, infection, and traumatic injury.

DEX has been well-documented to inhibit inflammatory response and reduce cytokine secretion in various experimental and clinical settings (Taniguchi et al., 2004; Memis et al., 2007; Bekker et al., 2013; Kang et al., 2013). However, the present study found that plasma CRP and TNF-α levels began rising within 24 h after surgery, peaked after 48 h, and began to decline at 72 h in both treatment groups; there were no intergroup differences. This inconsistency may be explained as follows. First, Zhang et al. (2012) demonstrated that DEX administration before ischemia, but not after, attenuated intestinal injury induced by intestinal ischemia/reperfusion. Thus, the inability of DEX to inhibit inflammatory responses in the present study may be due to already existed intestinal mucosal barrier damage caused by the soapsuds enema before DEX administration. Second, Zhang et al. (2012) also demonstrated that DEX dose-dependently produced intestinal protective effects as higher doses generated more obvious protective effects. However, the total dose of DEX used in the present study was below 1 μg/kg, which may not be adequate for suppressing LPS release and induction of downstream inflammatory responses. Third, determination of the appropriate sample size needed in the current study was calculated using duration to first flatus and may still lack the statistical power to detect differences in inflammatory indices between the treatment groups.

The identification of perioperative factors associated with duration to first flatus could provide insight into postoperative GI function recovery. Similar as our results, age and body mass index were reported to be independent risk factors for the development of postoperative paralytic ileus after radical cystectomy (Svatek et al., 2010). The possible reasons for an association of age with postoperative GI function recovery include decreased gastrointestinal motility, decreased mobility, and decreased narcotic tolerance in elderly patients (Svatek et al., 2010). Svatek et al. (2010)’s explanation for the association of BMI and postoperative ileus is a decrease or delay in ambulation after major surgery in obese patients compared with normal weight patients. However, the association of BMI with postoperative ileus was reported only to be specific for those patients with obesity and was not observed for overweight patients (BMI 25.0–29.9 kg/m^2^) (Svatek et al., 2010). Murphy et al. (2016) oppositely revealed that weight loss was an independent risk factor for developing postoperative ileus with a normal BMI. BMI of most patients recruited in our study was within normal range (below 29.9 kg/m^2^), thus we observed a negative association of BMI with duration to first flatus. Our results also indicated that gender and operative segments were associated with duration to first flatus. Male gender was reported to be an independent risk factor for postoperative ileus in anterior lumber interbody fusion in the elderly (Horowitz et al., 2018). Surgeons should take the increased risks of postoperative ileus into account when selecting a surgical approach for a male patient. Fineberg et al. (Fineberg et al., 2014) also suggested that male gender and 3+ fusion levels were independent predictors of postoperative ileus in lumbar fusions, that was consistent with our results.

Side effects of DEX are mainly restricted to hemodynamic alterations. These alterations include hypertension, bradycardia, and hypotension owing to pre- and postsynaptic α_2_-adrenoreceptor activation, which causes vasoconstriction, vasodilation, and reflex bradycardia. With a low dose of DEX, the present study found that the incidence of bradycardia in DEX group patients was even lower than in the control.

There were several possible limitations to the present study. First, only duration to first flatus was used to evaluate recovery of GI function. Duration to first flatus was reported by patients, and it may be relatively unreliable. Although no single objective variable has yet been found to accurately predict GI function recovery, future studies should employ combined functional outcomes of normalization of food intake and bowel function to adequately define this parameter. In addition, only one low dosage of DEX was employed to investigate the relationship between inhibitions of surgical stress and enhanced GI function recovery. More doses should be used to further investigate this relationship in future.

## Conclusion

The present study revealed that administration of low-dose DEX during surgery can accelerate GI function recovery in patients undergoing lumbar spinal surgery. This effect might partially be due to DEX’s ability to reduce postoperative opioid consumption rather than attenuation of inflammatory responses. The present results provide new insights for postoperative GI function recovery, showing that age, gender, BMI, operative segments, and overall sufentanil consumption significantly correlated with duration to first flatus.

## Data Availability Statement

All datasets generated for this study are included in the article/supplementary material.

## Ethics Statement

The studies involving human participants were reviewed and approved by Ethics Committee of Xuan Wu Hospital, Capital Medical University (Beijing, China). The patients/participants provided their written informed consent to participate in this study.

## Author Contributions

ML: Design and initiation of the study, patient recruitment, monitoring of processes, compilation of the CRF database, statistical analyses, composition of the first draft of the manuscript and preparation of the figures. TW: Design and initiation of the study, patient recruitment, monitoring of processes, compilation of the CRF database, statistical analyses, composition of the first draft of the manuscript and preparation of the figures. WX: Study conception and design, data acquisition and interpretation, article drafting, and critical review of the article. LZ and DY: contributed to the interpretation of the data, critical review of the article. Revision and approval the final version of the paper: all authors.

## Funding

This study was supported by Beijing Municipal Administration of Hospitals Incubating Program (CODE:PX2019031), Beijing Municipal Administration of Hospitals Clinical Medicine Development of Special Funding Support (ZYLX201818) and Beijing Municipal Administration of Hospitals Clinical Medicine Development of Special Funding Support (ZYLX201706).

## Conflict of Interest

The authors declare that the research was conducted in the absence of any commercial or financial relationships that could be construed as a potential conflict of interest.
